# Impact of climate change and management strategies on water and salt balance of the polders and islands in the Ganges delta

**DOI:** 10.1038/s41598-021-86206-1

**Published:** 2021-03-29

**Authors:** Mohammed Mainuddin, Fazlul Karim, Donald S. Gaydon, John M. Kirby

**Affiliations:** 1 CSIRO Land and Water, Canberra, ACT 2601 Australia; 2 CSIRO Agriculture and Food, Brisbane, QLD 4102 Australia

**Keywords:** Hydrology, Climate change, Climate and Earth system modelling

## Abstract

Enhancing crop production, particularly by growing a crop in the typically-fallow dry season is a key strategy for alleviating poverty in the Ganges delta region. We used a polder water and salt balance model to examine the impact of several crop management, salt management and climate change scenarios on salinity and crop evapotranspiration at Dacope and Amtali in Bangladesh and Gosaba in India. A key (and unsurprising) finding is that salt management is very important, particularly at the two drier sites, Dacope and Gosaba. Good salt management lowers salinity in the shallow groundwater, soil and water storage ponds, and leads to more irrigation. Climate change is projected to alter rainfall, and this in turn leads to modelled increases or decreases in runoff from the polders, and thence affect salt concentrations in the soil and ponds and canals. Thus, the main impacts of climate change are through the indirect impacts on salt concentrations, rather than the direct impacts of the amount of water supplied as rainfall. Management practices to remove salt from polders are therefore likely to be effective in combatting the impacts of projected climate change particularly at Dacope and Gosaba.

## Introduction

Globally, about 40 percent of the world population live within 100 km from the coastline^1,2^ and about 600 million people inhabit low-elevation coastal zones that will be affected by progressive salinization^3^. The Ganges delta includes a unique coastal zone of great significance for food security, biodiversity conservation, and fisheries production^4,5^. The coastal zone of the Ganges delta in Bangladesh includes 139 polders which are low-lying tracts of land surrounded by dikes constructed in the 1960s and 1970s to protect farming lands from saline water intrusion and tidal floods^6^. A less-well-developed polder system exists in the Sundarbans region of the West Bengal in India^7^. The ground level within polders is mostly within 0 to 3 m above mean sea level^8^. The level of water in the surrounding rivers rises and falls with the tidal flow and is affected by monsoon rain and occasional storm surge. This results in fluctuations in river water level of up to 4.0 m. The dense networks of canals (former river distributaries) within the polders are connected to the surrounding rivers by a series of sluice gates under the embankments providing the opportunity to bring water in during high tides, or to drain water from the polders during low tides, as needed. However, water management in the polders is poor, and infrastructures are inadequate, which makes the drainage of rain water accumulated within the polders difficult^4,7,9–11^. Due to insufficient drainage arrangement and high rainfall (~ 2000 mm, mostly concentrated during the monsoon season), polders are prone to flooding during the wet season. During the dry season the polder lands become salinised because of the shallow saline groundwater table and increasing salinity in the rivers as fresh water flows down the rivers decrease and the sea water moves further inland. About 65 percent of the lands of the coastal zones of Bangladesh and West Bengal are affected at various levels (low to high) due to salinity during the dry season^4,12,13^. Soil salinity is a major, and the most persistent, threat in the coastal zone of Bangladesh^14^ and Sundarbans region of West Bengal^7^.

The population of the salt-affected southwestern coastal zone of the Ganges Delta in Bangladesh is about 14.12 million (Barisal Division and Khulna, Bagerhat and Satkhira districts of Khulna Division^15^). In West Bengal, the population of the salinity-affected coastal zone called Sundarbans is 4.43 million^15^. These areas are disadvantaged by poverty, food insecurity, environmental vulnerability, and limited livelihood opportunities^5,16,17^. In West Bengal, 44 percent of the population is living below the poverty level and 87 percent people suffer from food shortages^7^. Similarly, in Bangladesh, 30.8 and 37.2 percent of the population of Khulna and Patuakhali districts, respectively, live below the poverty line compared with 24.3 percent for the whole country^15^. Agriculture is the principal livelihood activity in the Ganges delta and plays a key role in tackling the challenges of the growing population, alleviating poverty^18^ and maintaining food security^19,20^. The Governments of Bangladesh and West Bengal have developed comprehensive master plans for the agricultural development of the region to increase productivity and cropping intensity thus improving food security and livelihood^17,21^ of the population. Traditionally, farms produce low-yielding local rice varieties under rainfed conditions in the wet season^11,22–24^. In the dry season, most agricultural land remains fallow due to: (1) late wet season harvesting and prolonged waterlogging (which delays the planting of Rabi crops exposing the late planted crops to high soil salinity and untimely rains during the later part of the season), and (2) the lack (or perceived lack) of good quality irrigation water for Rabi season irrigation^9,11,23^. Recent experimental studies^24–30^ and modelling^8,9,31^ shown that crop production could be increased substantially with careful soil and crop management^9,29^. The key for such enhanced production is the management of water and salinity in the polders^9,31^ and their long-term sustainability due to impact of climate change^31^.

The impacts of global climate change on coastal agricultural production is very large across the world^32,33^ and the impacts have already begun to be visible in Bangladesh^20,34^. With increasing salinity problems due to sea level rise, coastal flooding and low freshwater flow, food production is largely hampered across the region^35–37^. The salt and water balance of a polder, and hence cropping opportunities are likely to be further affected by climate change^38,39^ and sea level rise^40,41^. Many of these issues have not been addressed in the previous studies. While there are some studies on quantifying climate impacts on coastal flooding (e.g.^42,43^) and salinity (e.g.^3,12,14,44–46^), polder scale salt balance were not investigated in those studies. Similarly, there are some studies on agricultural impacts of climate change in the coastal region or Bangladesh (e.g.^47–50^), these studies, however, have not incorporated management strategies in their analyses.

Our aim in this paper is to explore complex issues of increasing salinity and decreasing freshwater flows due to climate change and how the impacts could be minimized by adopting appropriate management strategies. The novelty of this study is to quantify the climate impacts on water and salt balance at a polder scale which has not previously been done. Another notable aspect of this study is to evaluate the impacts of individual management processes (e.g. dry season crops, groundwater pumping, reducing river water use, increasing the area of open water) on water salinity. We used a simple polder water and salt balance model developed by Mainuddin et al.^31^ to examine the direct and indirect impacts of climate change and salt and water management strategies on cropping in the polders. We show that the three polders exhibit similarities, but also differences that arise from both their different annual rainfall totals and from their differing salinities.

## Methods

### Location and characteristics of the polders

**Figure 1:**
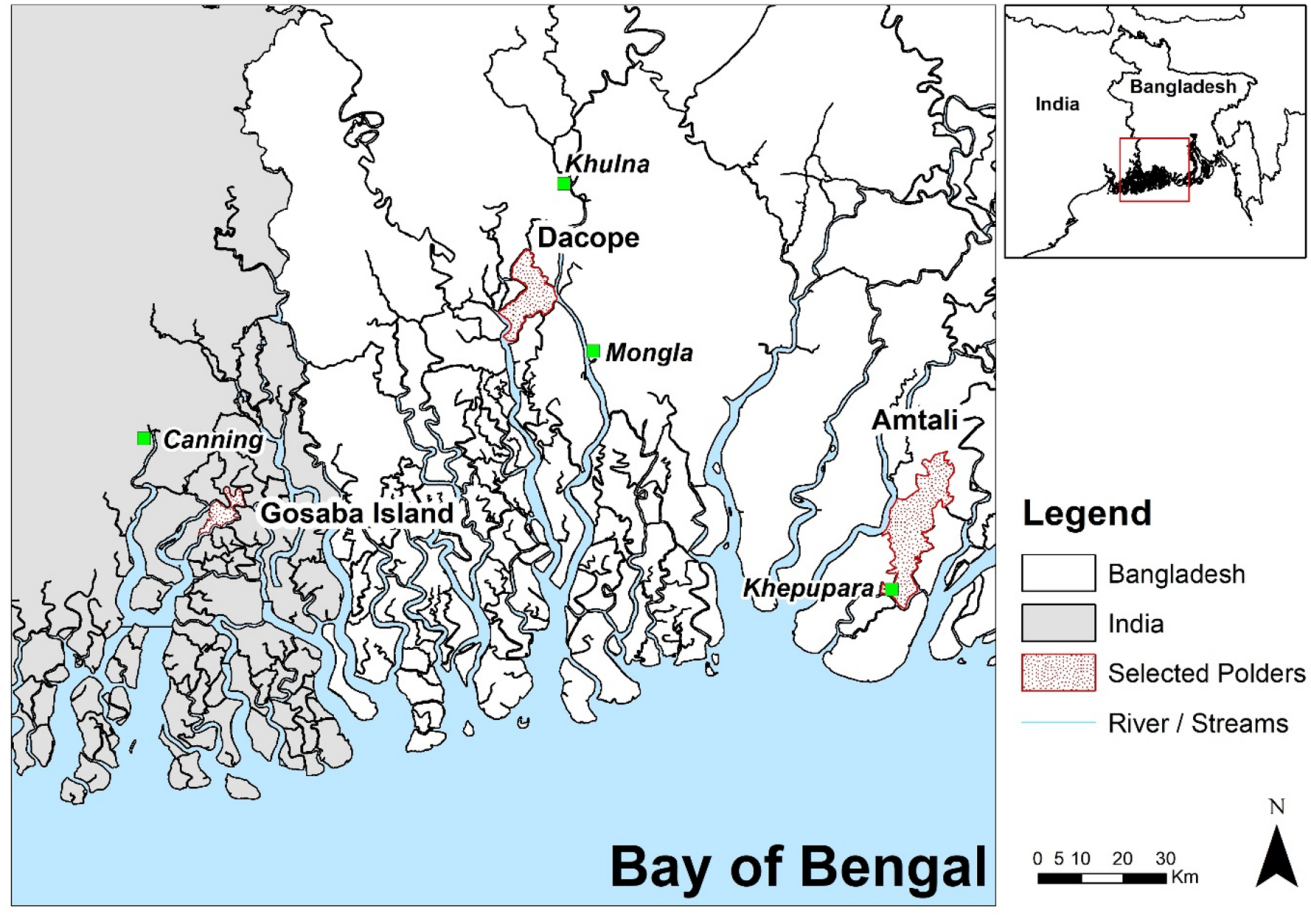
Study area map showing location of two polders (Amtali and Dacope) in Bangladesh and an island (Gosaba) in India (data source: https://data.humdata.org/dataset/bangladesh-water-courses).

Three study sites were selected for this study, two in Bangladesh and one in West Bengal, that include similarities and contrasts in salinity and water management options. These are Polder 43/1 in Amtali, Borguna (in the Patuakhali region) and the Polder 31 in Dacope, Khulna of Bangladesh and one island in the district of South 24 Parganas district of West Bengal (Fig. 1). The areas of the polders are 205 and 103 km^2^ for the Amtali and Dacope respectively. The area of Gosaba Island in West Bengal is 36 km^2^. The population density in these 3 polders/Islands are 548 person/km^2^, 1024 person/km^2^, and 831 person/km^2^, respectively for Amtali, Dacope and Gosaba^7,51,52^. The people depend mainly on agriculture for their livelihood^4,23^. Based on soil salinity, the polder at Amtali can be considered as low salinity, polder at Dacope as medium to high salinity and the Gosaba Island as high salinity, thus representing the range of salinities amongst the polders and islands of the Ganges Delta. The main characteristics of these 3 locations are compared in Table 1.

**Table 1:** Physical and hydro-climate characteristics of the three study sites.

Parameters	Amtali (Polder 43/1)	Dacope (Polder 31)	Gosaba Island
Area (km^2^)	205	103	36
Salinity and inundation level	Pronounced inundation in monsoon season but low to moderate salinity	Moderate to high salinity and prolonged inundation of low and medium land in monsoon season	High salinity and inundation vary with elevation within the Island
Annual Rainfall (mm)	2854	1854	1660
Mean daily maximum temperature (°C)	30.5	31.3	30.9
Mean daily minimum temperature (°C)	22.2	21.9	21.9
Annual potential evapotranspiration (mm)	1634	1733	1453
Water level in the surrounding rivers (m)	-0.5 to 2.5	-2.0 to 3.0	-0.5 to 3.0
Salinity of river water (g/L)	0.4 to 14	0.2 to 16	15 to 31
Groundwater depth (m)	0.8 to 1.8	0.04 to 1.09	1.0 to 2.5
Groundwater salinity (g/L)	1.9 (Nov/Dec) to 7.0 (May/Jun)	0.3 to 1.9	4.7 to 8.5

The climate of the Ganges delta is subtropical with observed annual average rainfall and potential evapotranspiration (PET) from 1994 to 2018 were, respectively about 2854 mm and 1634 mm at Amtali; 1854 mm and 1733 m at Dacope; and, 1660 mm and 1453 mm at Gosaba. About 82–83 percent of the annual rainfall occurs in the wet season during the month of May to October^53^. Average rainfall during December to February, which is the Rabi cropping season, is about only 5 percent of the total rainfall.

The salinity of the rivers surrounding the polders at Amtali and Dacope varies due to tidal condition and season. Salinity remains below 1 dS/m from August to December at Dacope and therefore considered suitable for irrigation for this part of the year. At Amtali, the salinity of river in the west side of the polder stays always at the acceptable level for irrigation. The salinity of the river in the east side of the polder increases after November and reaches its peak (about 25 dS/m) in May. After the onset of rainfall in June the salinity level of river water significantly decreases, and the river water becomes fresh from July to November. The salinity of the surrounding rivers in Gosaba Islands remains very high above 15 dS/m, so is unsuitable for irrigation throughout the year. There are dense networks of canals particularly in Bangladesh and also a lot of ponds. With proper management of the sluice gates and regulators, these canals and ponds can be used to store freshwater during the monsoon season for use in irrigation.

The average groundwater level at Amtali varies between 0.8 and 1.8 m from the surface and groundwater salinity varies from around 3 dS/m in November and December to 11 dS/m in May and June. Averaged groundwater level and salinity in Dacope varies between 0.04 to 1.09 m below the field surface and 0.4 to 2.9 dS/m, respectively. At Gosaba, the depth of groundwater is closest to the surface (+ 0.05 to -0.54 m) in August and September during the monsoon and the farthest (-1.08 to 1.93 m) during Rabi season. The mean salinity of the groundwater is about 10 dS/m ranging from 1.2 to 38.7 dS/m.

### Water and salt balance model

**Figure 2:**
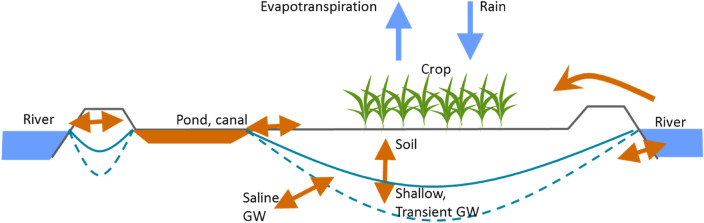
Schematic diagram of key processes governing water and salt balances in a polder. The blue arrows depict the movement of water without salt—rainfall and evapotranspiration. The brown arrows for the movement of water and salt between the rivers, groundwater, surface canals and ponds, and the soil. GW denotes groundwater.

The model and its calibration for the three study sites was described in detail by Mainuddin et al^9^. Here we give a brief description. The conceptual basis of the water and salt balance in a polder is shown in Fig. 2. The model uses a monthly time-step, and simulates the storage and movement of water and salt amongst three stores in the polder: a shallow transient groundwater lens sitting on top of an underlying saltier groundwater, the soil, and canal and ponds which may store and drain water (and hence salt) from the polder. Shallow freshwater lenses may be critical for crop production in coastal environments^54^, and are observed in the Ganges delta^55^. Water and salt also are exchanged between these stores and the external environment of the deeper, groundwater (presumed to be salty), the rivers that surround the polder, and the atmosphere. Salt is transferred with the water, except for rain and crop evapotranspiration which transfer water only. Simple process models govern the transfer of water (and hence salt). Crop evapotranspiration is calculated using a crop coefficient approach. When the soil is wet, water drains to the underlying shallow groundwater; when the soil is dry, water may be carried upwards by capillary rise. Excess rain is transferred as surface runoff to the ponds and canals; when the volume exceeds their capacity, the excess is drained to the rivers. When the water stored in the soil and available from capillary rise is insufficient for the crop, water may be transferred for irrigation from the river back to the canals and ponds and from there to the soil. The volume of water for irrigation is limited by the salt concentration of the water in the river and in the canals and ponds, and also by the volume of water stored in the canals and ponds. Finally, water (and salt) may flood the polder in storm surges.

Three instances of the model were calibrated by Mainuddin et al.^31^ for the three study locations which cover the range of water and salt environments in the delta (Table 1) from high rainfall / low evapotranspiration and low salinity at Amtali, to the lower rainfall/higher evapotranspiration and high salinity at Gosaba^31^. The calibration involved fitting the model to field observations such as canal and pond salinities, soil water salinity, shallow groundwater salinity and groundwater depth and also to the results of a 24-year time-series of crop water use and soil water and salt balance simulations using an independently validated APSIM (**A**gricultural **P**roduction **S**ystems s**IM**ulator ^56^) crop model. The latter comparison allowed the polder water and salt balance model to be calibrated to a longer time-series of results, and also tested parts of the model not directly addressed by calibration to the field results. The calibration of the polder water and salt balance model used one set of parameters at each site for both the comparison with the field measurements and the comparison with the APSIM model results. A sensitivity analysis on the model was also performed using a Monte-Carlo simulation. The results shows that polder water and salt behaviour are sensitive to parameters governing the transfer of salt and are most sensitive to a mixing parameter which governs how much the soil water (and hence its included salt) mixes with rain (or other) water that may fall on the soil and drain to the ponds and canals. Detail of model description and its calibration for the three study sites can be found in Mainuddin et al^31^.

### Input data

The input data are of three types: time series data, fixed parameters describing the polder soil and cropping system, and parameters which are designed to be varied to simulate scenarios. The time series data include monthly rainfall, potential evapotranspiration, and salt concentration. The fixed parameters include: the polder area; soil water storage and soil capillary rise rate parameters; cropping coefficients and crop salt sensitivity parameters (the latter govern the decline in crop evapotranspiration with increasing salt concentration in soil water).

Parameters which may be varied to simulate management strategies include: the area of ponds and canals (and the corresponding crop area) to allow more storage of water for irrigation; the depth to the regional groundwater, which may be varied to simulate groundwater pumping for salinity control and also to allow shallow aquifer storage of water for crop production; a salinity threshold above which canal or river water may not be used for irrigation, and a decision parameter (Yes / No) that determines whether irrigation is used or not.

### Climate change projections

The climate change scenarios consider results from IPCC’s 5th assessment report (AR5) based on 28 global climate models (GCMs, Table 2). The AR5 presents results from climate models of Phase 5 of the Coupled Model Intercomparison Project (CMIP5) along with greenhouse gas concentration scenarios termed as representative concentration pathways (RCPs). This study is based on projected future rainfall and PET under RCP4.5 scenario^57^. The GCM results were downscaled to the three study sites using an empirical downscaling or change-factor approach^58^.

**Table 2:** List of global climate models (GCMs), their founding institution and spatial resolution.

CMIP5 model ID	Founding institution	Country	Horizontal resolution (°lat x °long)
Access-1.0	CSIRO-BOM	Australia	1.9 × 1.2
Access-1.3	CSIRO-BOM	Australia	1.9 × 1.2
BCC-CSM1-1	Beijing Climate Center	China	2.8 × 2.8
BCC-CSM1-M	Beijing Climate Center	China	1.1 × 1.1
CanESM2	Canadian Centre for Climate Modelling and Analysis	Canada	2.8 × 2.8
CCSM4	National Center for Atmospheric Research	USA	1.2 × 0.9
CESM1-BGC	National Center for Atmospheric Research	USA	1.2 × 0.9
CESM1-CAM5	National Center for Atmospheric Research	USA	1.2 × 0.9
CNRM CM5	National Centre for Meteorological Research	France	1.4 × 1.4
CSIRO MK3-6	Commonwealth Scientific and Industrial Research Organisation	Australia	1.9 × 1.9
GFDL-CM3	Geophysical Fluid Dynamics Laboratory	USA	2.5 × 2.0
GFDL-ESM2G	Geophysical Fluid Dynamics Laboratory	USA	2.5 × 2.0
GFDL-ESM2M	Geophysical Fluid Dynamics Laboratory	USA	2.5 × 2.0
GISS-E2-H	NASA/Goddard Institute for Space Studies	USA	2.5 × 2.0
GISS-E2-H-CC	NASA/Goddard Institute for Space Studies	USA	1.0 × 1.0
GISS-E2-R	NASA/Goddard Institute for Space Studies	USA	2.5 × 2.0
GISS-E2-R-CC	NASA/Goddard Institute for Space Studies	USA	1.0 × 1.0
HadGEM2-AO	National Institute of Meteorological Research and Korea Meteorological Administration	Korea	1.9 × 1.2
HadGEM2-CC	Met Office Hadley Centre	UK	1.9 × 1.2
HadGEM2-ES	Met Office Hadley Centre	UK	1.9 × 1.2
INMCM4	Institute of Numerical Mathematics	Russia	2.0 × 1.5
IPSL-CM5A-LR	Institute Pierre Simon Laplace	France	3.7 × 1.9
IPSL-CM5A-MR	Institute Pierre Simon Laplace	France	2.5 × 1.3
MIROC5	Japan Agency for Marine-Earth Science and Technology (JAMSTEC)	Japan	1.4 × 1.4
MIROC-ESM	JAMSTEC	Japan	2.8 × 2.8
MIROC-ESM-CHEM	JAMSTEC	Japan	2.8 × 2.8
MRI-CGCM3	Meteorological Research Institute	Japan	1.1 × 1.1
NorESM1-M	Norwegian Climate Centre	Japan	2.5 × 1.9

**Figure 3:**
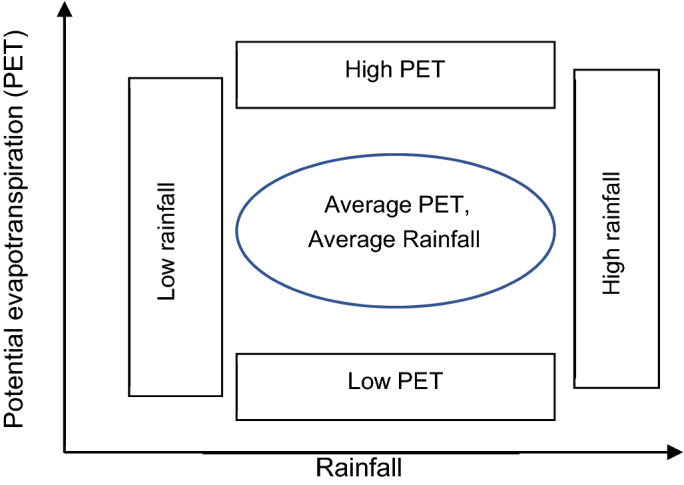
Schematic diagram of five climate scenarios by combining the low, average and high PET and rainfall.

**Figure 4:**
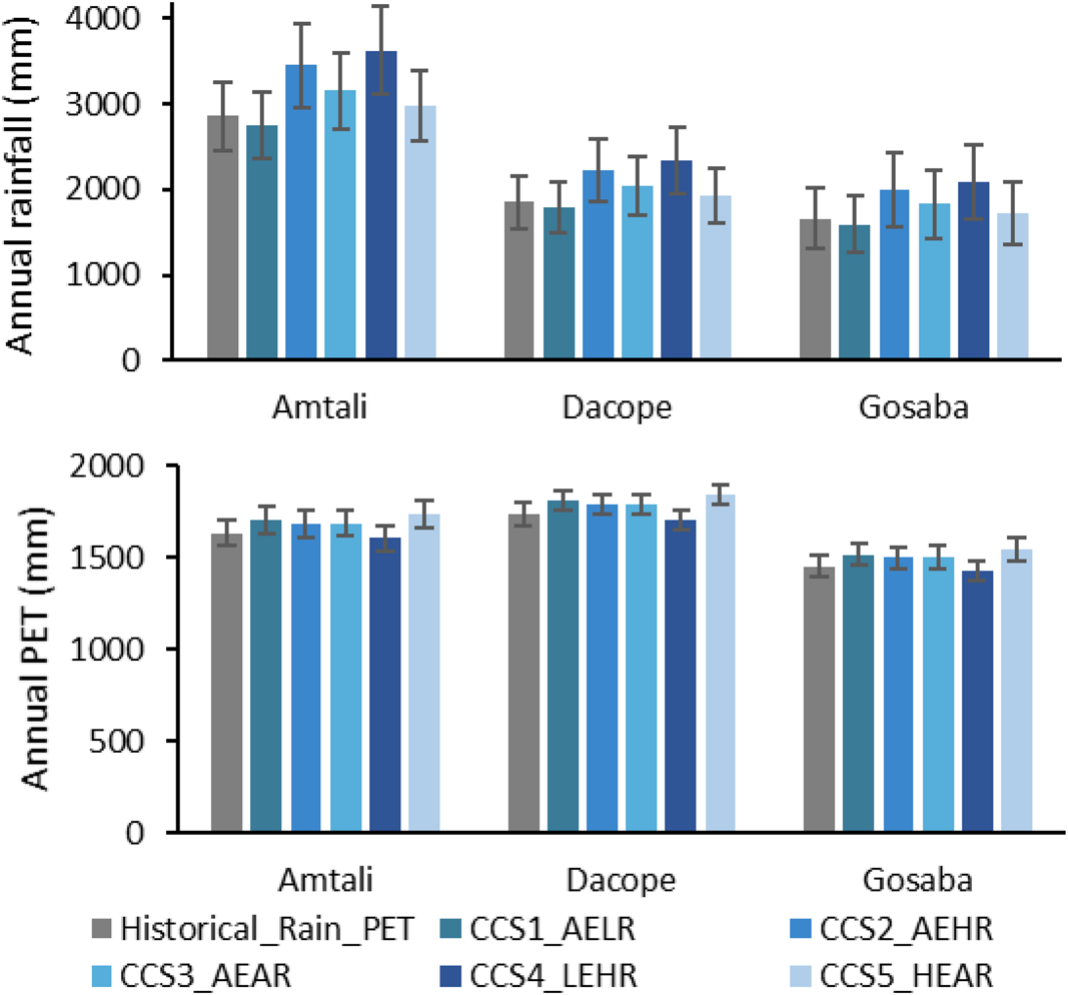
Comparison of the annual average rainfall (top) and potential evapotranspiration (PET, bottom) for the historical period and for the climate change scenarios (CCS1_AELR: average PET and low rainfall, CCS2_AEHR: average PET and high rainfall, CCS3_AEAR: average PET and average rainfall, CCS4_LEHR: low PET and high rainfall, CCS5_HEAR: high PET and average rainfall).

The scenarios were selected to represent the range of rainfall and PET projected by 28 GCMs. Five scenarios were considered, one for average of all models, two for rainfall extremes (low and high) and two for PET extremes (low and high) as shown in Fig. 3. Future climate projections from all 28 GCMs were investigated and a GCM was selected for each scenario that projected closest rainfall and PET to the average value of all GCMs. It is important to note that GCMs were selected considering combined impacts on rainfall and PET rather than considering each variable independently. This avoids artificially inflating the uncertainties by treating the uncertainties in rainfall and PET projections as independent. While we have selected five GCMs for use. The five scenarios are as follows: (1) average PET and low rainfall (CCS1_AELR), (2) average PET and high rainfall (CCS2_AEHR), (3) average PET and average rainfall and (CCS3_AEAR), (4) low PET and high rainfall and (CCS4_LEHR), and (5) high PET and average rainfall (CCS5_HEAR). Detail of climate scenario selection can be found in Karim et al^59^. Projections were made over the period of 2045 to 2075 for the three selected polders and the changes were estimated at annual, seasonal, and monthly time scale. We have used the period of 2046 to 2075 to represent the mid-term global climate change by 2060 as reported in Zheng et al^58^. Also, we have used 30 years predictions (2046–2075) to estimate mean change by 2060. This helps reducing uncertainty in GCM predictions. The annual average rainfall and the average annual potential evapotranspiration of the climate change scenarios are compared to the historical averages in Fig. 4.

### Management and climate scenarios

The calibrated polder models for Amtali, Dacope and Gosaba were used to simulate the salt and water dynamics of 17 scenarios for each polder. In each scenario, the model simulated a 25-year period, using the rainfall and potential evapotranspiration time series from 1994 to 2018 or, in the case of climate change scenarios, for a 25-year period representing projected climates for 2046 to 2070.

The salt concentration of the shallow, fresh groundwater lens changes slowly over several years in the polder model simulations. Thus, the salt concentration of the lens depends on the initial salt concentration as much as it is on the evolution of its behaviour over the simulation period. This slow evolution of the salt concentration can in turn affect the simulated salt concentration of other stores, such as the soil salt store. To provide a comparison between scenarios not influenced by the initial conditions, in all scenario simulations the polder model was run several times, with the shallow groundwater salinity averaged over the five years at the end of each iteration being used as the initial salt concentration for the next iteration. The model was run until the changes in salt concentration between iterations reduced to less than 0.1 percent. In some cases, the salt concentrations oscillated between two values (always values close together) in successive iterations, but the oscillation was itself stable over many iterations.

### Management scenarios

The current dominant cropping practice, rice—fallow rotation (R0), is considered as the base condition. For future cropping, five management scenarios were assessed. These scenarios were as follows:

(i) Rice—fallow rotation (R0), no Rabi crops. This scenario represents the base condition.
(ii) Rice—Rabi rotation, using wheat as a representative Rabi crop (denoted RW), with rice grown during the Kharif season (July to November) and wheat grown during the Rabi season (November to April), with periods of fallow between. The water requirement of wheat is representative to many other crops such as maize, sunflower, potato, tomato etc. which are grown in the region. Considering another crop would not have made any difference to the overall model results. Wheat is still grown in the region ^15,25,60,61^. The fallow periods have no transpiration from vegetation but do have soil evaporation. This scenario is the same as the calibration scenarios described by Mainuddin et al^31^.
(iii) Rice—Wheat rotation with groundwater pumping (RW-GP), which is the same as the second scenario except for some assumed groundwater pumping. The assumed groundwater pumping is designed to increase the depth to the saline water table underlying the shallow, fresher surface water table, so that more fresh water may be stored in the aquifer for crop use. This is also expected to prevent capillary rise of the saline groundwater to the rootzone of the crops grown in the Rabi season thus preventing salt impact to the crops. In the other simulations, the saline water table is assumed to be at a depth of 1.5 m, which resulted from the calibrations. In the groundwater pumping scenario, the depth is increased to beyond 4 m, the depth to which the water table fell in the simulations, and thus the deeper, salty groundwater had no effect on the simulated results. This modest depth of pumping is well within the depths to which groundwater routinely pumped in much of Bangladesh, and therefore unlikely to present technical or economic barriers in the polder setting. The salt and water pumped out of the shallow aquifer is assumed to be disposed of to the river without affecting the salt loads or water volumes in any other part of the system. In principle, this might be achieved either by pumping directly to the river or draining the pumped water to the river via the canal system with careful management to leave no residual salinity effects.
(iv) Rice—Wheat rotation with no irrigation allowed from river water (RW-NoR).
(v) Rice—Wheat rotation with a larger area of ponds and canals (RW-MP). The larger area allows for more stored pond and canal water to be used for irrigation (provided it remained at a salt concentration below the threshold allowed for irrigation). The area of ponds and canals was increased to 25 percent of the total area for Amtali and Gosaba (where the initial area was less than 10 percent of the total) and to 33 percent for Dacope (where the initial area was about 18 percent of the total).
(vi) Rice—Wheat rotation with irrigation allowed from river water (RW-RI), irrespective of the salt concentration in the river. Essentially, this is a simulation of the breakdown of careful control over the polder sluice gate and irrigation operations.

### Climate change scenarios

We first applied 5 climate change scenarios (scenarios vii to xi) described in Sect. 2.3 to the base case (R0-CCS1_AELR, R0-CCS2_AEHR, R0-CCS3_AEAR, R0-CCS4_LEHR, R0-CCS5_HEAR) to understand the impact of climate change in the area if the lands are kept as it is i.e. there is no cropping in the Rabi season in future. The management scenarios considered above would alter the salt and water balance in the polders. They can be further affected by the potential impacts of climate change in the future. So, to consider the combined impacts of dry season cropping and the future climate change following five scenarios are considered.

(xii). Rice—Wheat rotation impacted by climate change with an average changed evapotranspiration and a slight reduction in rainfall (RW-CCS1_AELR), based on the ccsm4 GCM.
(xiii) Rice—Wheat rotation impacted by climate change with an average changed evapotranspiration and large increase in rainfall (RW-CCS2_AEHR), based on the hadgem2 GCM.
(xiv). Rice—Wheat rotation impacted by climate change with an average changed evapotranspiration and average increase in rainfall (RW-CCS3_AEAR), based on the bcc-csm1 GCM.
(xv). Rice—Wheat rotation impacted by climate change with a small reduction in evapotranspiration and large increase in rainfall (RW-CCS4_LEHR), based on the giss-e2 GCM.
(xvi). Rice—Wheat rotation impacted by climate change with a large increase in evapotranspiration and average increase in rainfall (RW-CCS5_HEAR), based on the gfdl-cm3 GCM.

The final scenario (xvii) is that of an increase in river salinity that results from the rise in sea level that is projected to accompany climate change. In the present day, low flow during the dry season leads to sea water intruding up the rivers of the Ganges delta. The effect is projected to be greater with sea level rise^3,12,44,62^. The magnitude of the salinity increase will depend on the balance of sea level rise and the supply of fresh water coming down the rivers^44^. With the greater river flows in the eastern part of the delta, the increases in river salinity are likely to be less there^44^. In contrast, Akter et al.^12^ suggested that the eastern part of the delta will experience large (“alarming”) increases in salinity. In the polder model, the monthly time-series of river salinity is an input, with water fresher in the wet season than the dry season. We scaled the monthly values to increase the average salt concentration by 5 g/L. This increase may be more at Amtali (in the east of the delta) than is suggested by Bricheno and Wolf ^44^ and is more in line with the suggestion of Akter et al.^12^ in any event it allows us to easily compare the increase in the three locations.

- (xvii) Rice—Wheat rotation impacted by increased salinity in the rivers resulting from sea level rise (RW-Sr +).

Implicit in this final scenario is that the management of irrigation and drainage water in and out of the polder is not impeded by higher river water levels which result from the sea level rise. While there may be some greater difficulty in drainage, this can in principle be accomplished by installing pumps. The consequences of a breakdown in proper drainage management are examined in scenario (vi).

## Results

### Comparison of baseline condition of the 3 polders

**Figure 5:**
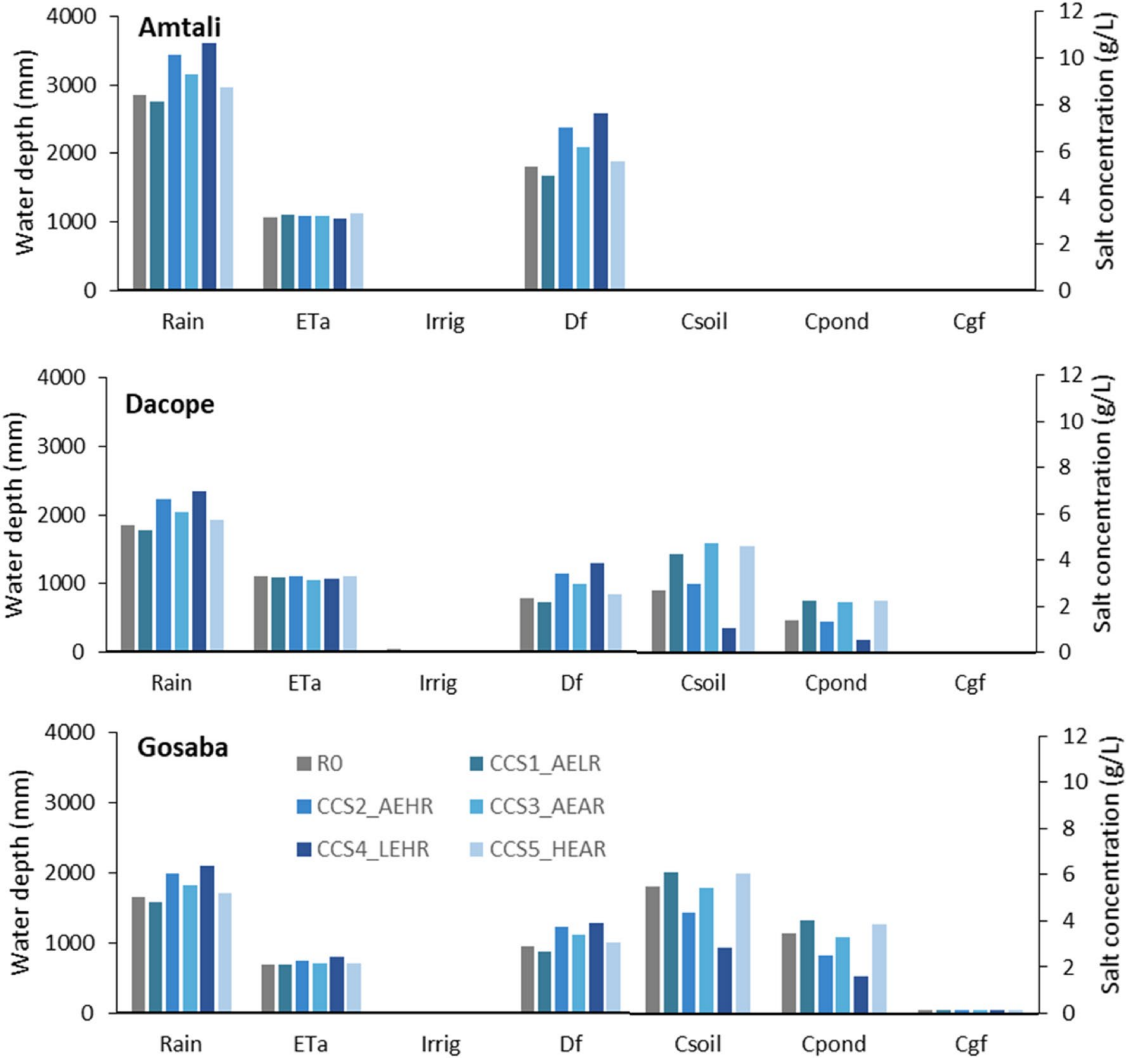
Comparison of water and salt balance for the base case rice-fallow management and for 5 climate scenarios for Amtali (top), Dacope (middle) and Gosaba (bottom), (base case scenarios i and scenarios vii to xi). Each group of columns shows the average annual water depth (left axis) and salt concentration (right axis). The average annual water depths are rain, actual evapotranspiration (ETa), applied irrigation (Irrig), and surface drainage from the fields which is equivalent to runoff from the soil to the canals and ponds (Df). The salt concentrations are concentration in the soil water (Csoil), in the pond water (Cpond) and in the fresh shallow groundwater (Cgf).

The observed annual average rainfall from 1994 to 2018 at Amtali was 2854 mm, whereas Dacope had 1854 mm and Gosaba had 1660 mm. As expected from the greater rainfall, Amtali shows by far the greatest runoff from the surface field drainage (Fig. 5). Gosaba is simulated to have the least irrigation, because in most scenarios the sources of irrigation water (the river and the canals and ponds) are too salty. Amtali has little irrigation because it needs little, owing to the higher rainfall some of which is stored in the soil and available to crops in the dry season. Dacope has the most irrigation, because of its greater need combined with greater availability than Gosaba (Fig. 5).

Amtali is simulated to have the least salty soil, canals and ponds, and shallow groundwater. Dacope is intermediate, and Gosaba has the saltiest soil, canals and ponds, and shallow groundwater.

### Impacts of climate change

Climate change is projected to affect both the water balance and the salt balance in the polders. Higher rainfall is projected to lead to greater runoff (field surface drainage, denoted Df in Fig. 5) and lower salt concentrations at all three sites, whereas lower rainfall leads to lesser runoff and higher salt concentrations.

The changes (%) in water balance components are summarised in Table 3. There are significant increases in rainfall for 3 scenarios (R0-CCS2_AEHR, R0-CCS3_AEAR, R0-CCS4_LEHR) which increased surface drainage (Df) in all 3 sites.

**Table 3:** Impact of projected future climate on rainfall (Rain), actual evapotranspiration (ETa) and surface runoff (Df).

Scenarios	Changes (%) with respect to baseline condition (R0)								
	Amtali			Dacope			Gosaba		
	Rain	ETa	Df	Rain	ETa	Df	Rain	ETa	Df
R0-CCS1_AELR	− 3	4	− 7	− 4	− 2	− 8	− 4	1	− 7
R0-CCS2_AEHR	21	2	32	20	− 1	46	20	7	30
R0-CCS3_AEAR	11	2	16	10	− 5	27	10	1	17
R0-CCS4_LEHR	27	− 1	43	26	− 4	64	26	16	34
R0-CCS5_HEAR	4	5	4	4	0	7	4	2	5

### Impacts of management scenarios

#### Dry season crop

**Figure 6:**
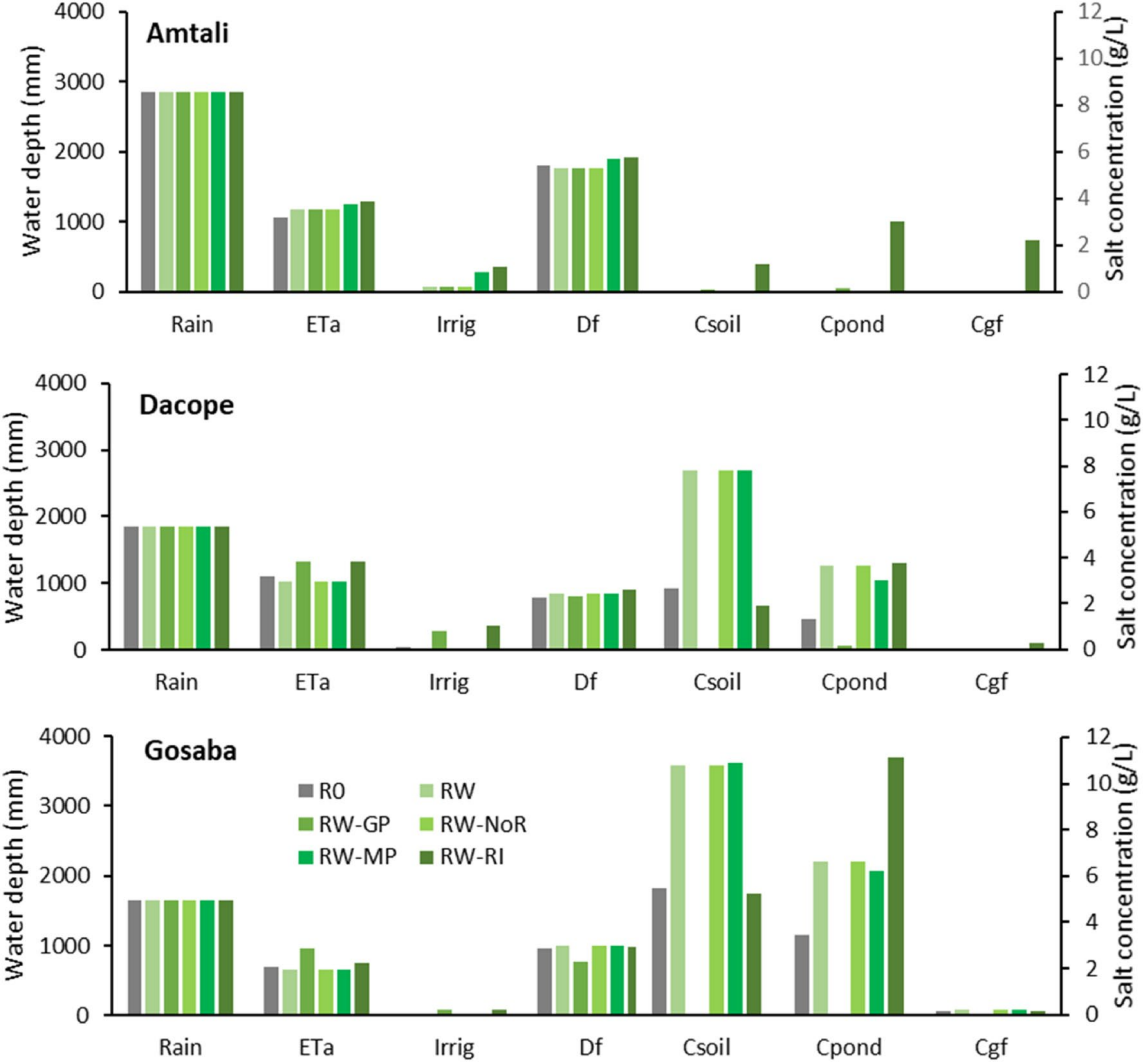
Comparison of water and salt balance for 5 land management scenarios for the Amtali (top) and Dacope (middle) polders and Gosaba Island (bottom) (base case scenarios i and scenarios ii to vi). Each group of columns shows the average annual water depth (left axis) and salt concentration (right axis). The average annual water depths are rain, actual evapotranspiration (ETa), applied irrigation (Irrig), and surface drainage from the fields which is equivalent to runoff from the soil to the canals and ponds (Df). The salt concentrations are concentration in the soil water (Csoil), in the pond water (Cpond) and in the fresh shallow groundwater (Cgf).

The difference between the rice–wheat (RW scenario in Fig. 6) and rice—fallow (RO) rotations is seen in the simulated lower evapotranspiration of the rice—fallow at Amtali. This in turn leads to somewhat increased runoff. The greater uptake of groundwater with a dry season crop at Dacope and Gosaba leads to a saltier soil, which in turn leads to saltier ponds and canals. At Dacope and Gosaba there is no irrigation, and the saltier soil of the rice—wheat rotation depresses the evapotranspiration.

#### Groundwater pumping

Groundwater pumping (RW-GP scenario in Fig. 6) results in smaller simulated salt concentrations in the soil and the ponds at Dacope and Gosaba. (The salt concentrations at Amtali are low to begin with, and not much affected by the pumping). This is because of the movement of salt by capillary rise from the saline deeper groundwater into the soil, and thence via surface field drainage to the ponds, is reduced in this scenario. At Dacope and Gosaba, the less salty ponds and canals leads to them being used for irrigation, which in turn leads to an increase in the evapotranspiration. This scenario is based on the assumption that the deep, salty groundwater is disposed of directly into the river, and not into the ponds and canals; disposal into the ponds and canals would re-introduce the salt back into the soil via the irrigation water.

#### Stopping the use of river water

Stopping the use of river water for irrigation (RW-NoR scenario in Fig. 6) generally results in little simulated difference with the base rice–wheat scenario, because the base scenario made little use of river water anyway.

#### Increasing the ponds and canals

The increased area of ponds and canals (scenario RW-MP in Fig. 6) was simulated to lead to greater use of irrigation at Amtali. However, the simulated impact at Dacope and Gosaba is limited compared to the RW scenario, because the ponds and canals are generally too salty to use for irrigation.

#### Using river water for irrigation

This scenario (RW-RI in Fig. 6) is essentially a simulation of the breakdown of careful control over the polder sluice gate and irrigation operations. The salt concentrations in some or all of the soil, the ponds and canals, and the groundwater was found to rise at all three locations. The impact on groundwater is significant only at Amtali. At Gosaba, the simulated soil salinity decreases with the forced use of river water, and the evapotranspiration increases. This is because the river water (the salt concentration of which is diluted by the water in the ponds and canals) results in more water becoming available for irrigation, which substitutes for the uptake of salty groundwater.

The impacts of management scenarios on water balance are summarised in Table 4. Due to cropping, there are changes in actual evapotranspiration. There is not much change in drainage.

**Table 4:** Changes (%) in rainfall (Rain), actual evapotranspiration (ETa), and surface runoff (Df) under different management scenarios.

Scenarios	Changes (%) with respect to baseline condition (R0)								
	Amtali			Dacope			Gosaba		
	Rain	ETa	Df	Rain	ETa	Df	Rain	ETa	Df
RW	0	10	− 2	0	− 7	8	0	− 6	4
RW-GP	0	10	− 2	0	19	3	0	37	− 19
RW-NoR	0	10	− 2	0	− 7	8	0	− 6	4
RW-MP	0	18	5	0	− 7	8	0	− 6	5
RW-RI	0	21	7	0	19	14	0	9	3

#### Impacts of combined management and climate scenarios

**Figure 7:**
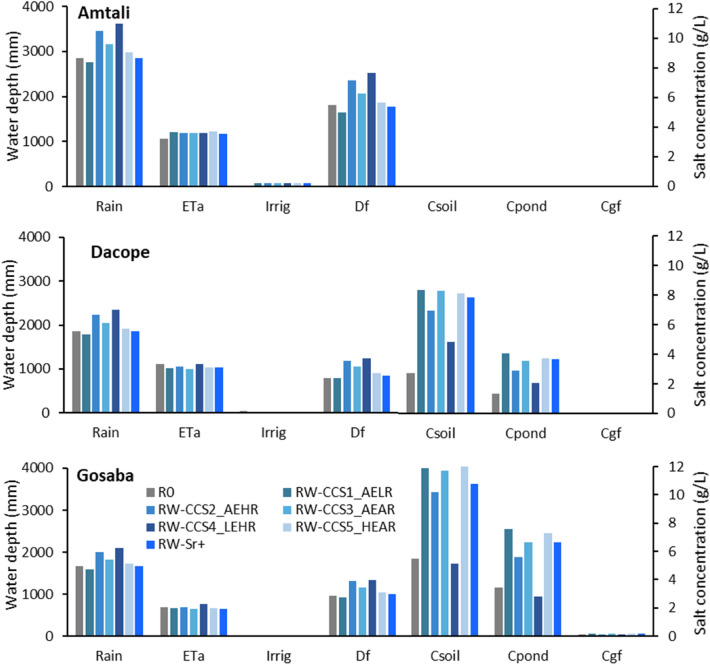
Comparison of water (left hand axis) and salt (right hand axis) balance under combined effects of land management and climate scenarios for the Amtali (top) and Dacope (middle) polders and Gosaba Island (bottom) (base case scenarios i and scenarios xii to xvii).

The combined effects of land management and climate change on the water balance and the salt balance are highly pronounced in all three polders with the two highest rainfall projections (scenarios RW-CCAEHR and RW-CCLEHR) leading to the greatest runoff (Fig. 7). The salt concentrations at Dacope and Gosaba are about double those found in the climate change scenarios under rice—fallow. At Gosaba, the high rainfall—low evaporation scenario leads to projected lower soil and pond salt concentrations than in the base case, but the site is nevertheless too salty for irrigation. At Dacope and Gosaba, the other climate change scenarios lead to higher soil and pond salt concentrations than in the base case. The salt concentrations at Amtali remains low in all climate change scenarios. The increasing salinity in the rivers combined with a rice—wheat management system is projected to have an impact similar to that of rice–wheat combined with climate change.

The impacts of combined land management and climate scenarios on major water balance parameters are summarised in Table 5. There is noticeable change in rainfall, actual evapotranspiration, and surface drainage due to the impact of climate change on the land management scenarios.

**Table 5:** Changes (%) in rainfall, actual evapotranspiration (ETa) and surface runoff (Df) in the coastal polders under combined effects of climate change and land management scenarios.

Scenarios	Changes (%) with respect to baseline condition (R0)								
	Amtali			Dacope			Gosaba		
	Rain	ETa	Df	Rain	ETa	Df	Rain	ETa	Df
RW-CCS1_AELR	− 3	13	− 8	− 4	− 9	1	− 4	− 5	− 3
RW-CCS2_AEHR	21	12	31	20	− 5	51	20	− 2	37
RW-CCS3_AEAR	11	11	15	10	− 10	35	10	− 6	22
RW-CCS4_LEHR	27	11	40	26	0	57	26	10	39
RW-CCS5_HEAR	4	14	3	4	− 6	15	4	− 4	10
RW-Sr +	0	10	− 2	0	− 8	8	0	− 6	4

## Discussion

The results of the management scenarios show that the biggest impacts on the salt concentrations of the soil, ponds and canals and shallow groundwater lens result from the management strategy to lower the salty deeper groundwater table and from the management failure of allowing salty river water to be used for irrigation. At Gosaba, these strategies also have the biggest impact on the crop evapotranspiration, and on the availability of water at a low enough salt concentration for irrigation. In contrast, the strategy to increase the area of ponds and canals for rice–wheat production provides more water but does not much affect the salt concentrations compared to the rice–wheat system without more ponds. The impact of this strategy on salt concentrations and the crop evapotranspiration is small. Mainuddin et al.^31^ came to a similar conclusion based on a Monte-Carlo parameter sensitivity analysis. They did not examine management scenarios as we have done here; rather they examined the sensitivity to variation in several input parameters including soil and groundwater properties and soil, groundwater, and river salinity. Their key finding was that, in general, the impacts were more sensitive to parameters governing the transfers of salt, and less sensitive to those governing the availability of water. The crop evapotranspiration was particularly sensitive to the model parameters. The implication is that management (including drainage) to remove salt is likely to have a greater impact than strategies to improve water availability.

The base case (rice-fallow) climate change results show that the biggest impacts on salt concentrations arose from the result of the two higher rainfall scenarios. The higher rainfall suppressed the need for water to be supplied from saltier sources of groundwater and the canals and ponds, and also flushes more salt out of the system. The less salty environment thus produced at Gosaba leads to greater crop evapotranspiration. The evapotranspiration effect is not seen at Amtali and Dacope; they are less salty in the first place, and so reducing the salt concentrations did not much affect the evapotranspiration. Thus, the impact of the projected climate changes is largely through the impact on salt concentrations, rather than the change to water availability. Furthermore, the impacts are greater at the driest and saltiest location, Gosaba. Payo et al.^46^ showed that under climate change scenarios the area of no-salinity is likely to decline, but it will decline much less under a wetter scenario than a drier one. Our results agree with the conclusion that drier scenarios lead to saltier outcomes but, since we take account of factors which they do not, we find that wetter scenarios lead to less salty outcomes than a no climate change scenario.

The combined climate change and management change results are similar to those of the base case climate change results. However, the potential impact of the additional wheat crop resulting from the greater potential evapotranspiration is offset by greater salt concentrations in the soil suppressing additional evapotranspiration. The soil and canals and ponds at both Dacope and, particularly, Gosaba become saltier under the combined climate change and management change than is the case for the rice-fallow climate change scenarios. At Amtali, the salinity of the soil and canals and ponds remains low in all cases.

The features above suggest some practical considerations for polder management. Firstly, these results suggest that providing additional water through increasing water storage capacity of the soil or increasing surface water storage (canal and ponds) is not critical ^31^. This follows from the fact that at all three sites, the evapotranspiration is less than rainfall and most of the crop needs are supplied by the combined water storage of the soil and shallow groundwater. Schulthess et al.^61^ also found that irrigation had little impact on maize and no impact on wheat in the same area. The impact was significant only in the driest years of their trials ^61^. However, the polder model is not a detailed crop model and is not responsive to the timing of irrigation events, some of which may be important^31^.

Secondly, several management strategies are available to manage salt, which is a more critical factor than the water availability. The strategies include subsurface field drainage, groundwater management (lowering of salty groundwater tables), and the management of the canals and ponds to maximise the export of salt from the polder and minimise its import. The drainage and salt management of Dutch polders^63^, for example, is done through a highly engineered and managed system than is practised in the Ganges delta. It is of course unsurprising that salt management is critical. Salt import into the soil depresses crop production, while salt export enhances it.

Another issue that cannot be modelled with the polder model is waterlogging impacts. The increased rain in some climate change scenarios might lead to more waterlogging problems, with adverse impacts on crop production. Again, this issue is properly addressed by a crop production model such as APSIM. However, field drainage as discussed above for salt management is also likely to be effective for waterlogging management.

In contradiction of this result is that irrigation has proven to be useful in the field experiments^9,29^. But irrigation will involve the addition of salt as well as water to the soil surface; the amount will depend on the salt concentration in the ponds and canals. Without adequate drainage, some of the added salt is likely to be leached downward in the wet season to the water table, only to be brought to the surface again by capillary rise in the next dry season. This recycling of salt would, over several years, lead to high salt concentrations that could limit crop growth. What is not shown by the field experiments is how much salt is exported from the small field experimental plots and, conversely, how much salt is stored in the soil and groundwater in and under the plot. The experiments therefore do not offer much information on the long-term sustainability of the irrigation practice. Enhancing the removal of salt will enhance the long-term sustainability.

For enhancing the removal of salts, we considered a scenario of pumping saline groundwater in the dry season. Pumping will lower the groundwater table which will not only remove salt from the soil but also will reduce the problem of water logging^64^. There is extensive groundwater extraction for irrigation in the other regions of Bangladesh^65,66^. The cost of pumping groundwater is very high. For example, for Boro rice cultivation the irrigation costs about one fourth of the total cost of production^65^. Notwithstanding the high cost of pumping, farming elsewhere in Bangladesh is profitable. In this study, we wanted to test the technical feasibility of groundwater pumping for drainage to grow crops in the dry season. We did not analyse the economic profitability. However, depending on the type of crops grown in the dry season, market, and other conditions, it is reasonable to expect that pumping of groundwater could be profitable in the future, as it is elsewhere in Bangladesh.

The population of Bangladesh is projective to increase to 202 million in 2050 from the current 168 million^67^. This will require about 10 million tonnes of additional rice production, the main staple food for the people^20^. The deficit of the other major grain, wheat, will grow to about 6 million tonnes by 2050^20^. So the country must keep producing additional food from a land-area, mostly excluding the coastal zone, that is declining at a rate of 1percent per year due to urbanization and industrial development^68,69^. In response, the Government of Bangladesh is giving the utmost importance to the agricultural development of the coastal zone^17^ and has identified the area as a hotspot for development in the recent Bangladesh Delta Plan 2100^70^. Similar importance is being given by the Government of West Bengal for the development of the Sundarbans. Thus, the results of this study are expected to provide valuable information for the policy makers and planners of the respective governments.

The results suggest the impacts of sea level rise are not particularly great. This contrasts with other studies (e.g.^3,12,44,62^) that suggest that the impacts are likely to be large. The difference arises because in our study, the saltier river water is not used, either because not much is needed (Amtali) or the river is already too salty for use (Gosaba). There is a modest impact at Dacope, though it is still less than suggested by the papers noted above. We have not explicitly modelled the impact of a rise in sea level on drainage of the polders, which may be more difficult to manage effectively. However, the consequences of a breakdown in effective drainage and irrigation management was seen in scenario 6, which generally increased salt concentrations in the polder. Thus, the impacts of a rise in sea level, and hence river salinity and river levels appear to be primarily through the consequences for drainage management. In principle, this can be managed by appropriate infrastructure, perhaps including pumps.

A major limitation of the assessment described in this paper is that we have not considered the impact of sea level rise on the flooding and consequent changes in salt additions to polders. Haque et al.^62^ show that river, tidal and storm surge flooding is likely to increase in the future due to climate change and sea level rise; however, the maintenance and improvement of polder embankments may offset the tendency to more flood. Flooding of polder by saline water during storm events and the subsequent water and salt dynamics is simulated by the polder model and will be considered in another paper.

## Conclusions

The main consideration resulting from the understanding of the polder salt and water balance processes and the model is the importance of strategies to remove salt. These strategies lead to somewhat higher crop evapotranspiration which, all other things being equal, would result in greater crop production. They also lead to long-term sustainability. Lowering of salty groundwater table, the provision of field drainage to reduce soil salinity, and the management of larger polder drainage canals to remove salt from the polder are all likely to be effective.

While the direct impacts of projected climate changes are to alter the amount of water added to or removed from the polder, this indirectly impacts the removal of salt in the runoff and drainage, and on the requirement to use other, saltier sources of water to satisfy crop water requirements. Thus, salt management strategies are also likely to be important in combatting the impacts of climate change.

Overall, the impacts of the various scenarios were found to have least impact at Amtali and Gosaba, in the east and west of the delta. The former has the greatest rainfall and least potential evapotranspiration, freshest water in the surrounding rivers, hence the least irrigation requirement with the greatest potential external supply of water for irrigation, and the greatest scope for flushing salt out. The latter has lower rainfall and greater potential evapotranspiration, the saltiest river water, hence a greater irrigation requirement but with little scope to satisfy it, and the least scope for flushing salt out from agricultural lands. Dacope, which is intermediate in terms of salinity in river water, shows more impact of the scenarios since there is some scope for irrigation and flushing salt out and the opportunities are sensitive to the scenarios.
